# Tipping points ahead? How laypeople respond to linear versus nonlinear climate change predictions

**DOI:** 10.1007/s10584-022-03459-z

**Published:** 2022-11-21

**Authors:** Felix J. Formanski, Marcel M. Pein, David D. Loschelder, John-Oliver Engler, Onno Husen, Johann M. Majer

**Affiliations:** 1 Faculty of Sustainability, Leuphana University Lüneburg, Universitätsallee 1, 21335 Lüneburg, Germany; 2 Faculty of Business and Economics, Leuphana University Lüneburg, Lüneburg, Germany; 3 Faculty of Natural and Social Sciences, University of Vechta, Vechta, Germany; 4 Faculty of Education and Social Sciences, University of Münster, Münster, Germany; 5 Faculty of Education and Social Sciences, University of Hildesheim, Universitätsplatz 1, 31141 Hildesheim, Germany

**Keywords:** Climate change communication, Risk perception, Tipping points, Abrupt climate change

## Abstract

We investigate whether communication strategies that portray climate change as a nonlinear phenomenon provoke increases in laypeople’s climate change risk perceptions. In a high-powered, preregistered online experiment, participants were exposed to linear or nonlinear predictions of future temperature increases that would be expected if global greenhouse gas emissions were not reduced. We hypothesized that the type of climate change portrayal would impact perceptions of qualitative risk characteristics (catastrophic potential, controllability of consequences) which would, in turn, affect laypeople’s holistic risk perceptions. The results of the study indicate that the type of climate change portrayal did not affect perceptions of risk or other social-cognitive variables such as efficacy beliefs. While participants who were exposed to a nonlinear portrayal of climate change perceived abrupt changes in the climate system as more likely, they did not perceive the consequences of climate change as less controllable or more catastrophic. Notably, however, participants who had been exposed to a linear or nonlinear portrayal of climate change were willing to donate more money to environmental organizations than participants who had not been presented with a climate-related message. Limitations of the present study and directions for future research are discussed.

## Introduction

Although global temperatures have risen at an alarming rate in recent decades (IPCC 2021), policymakers around the world have been hesitant to initiate large-scale efforts to combat climate change. Science communicators have long attempted to overcome this widespread hesitancy in the face of climate change by highlighting the risks of wait-and-see-approaches to climate policy (e.g., Moser 2010; Russill and Nyssa 2009). Reviews suggest that since the mid-2000s, communicators have increasingly relied on communication strategies that stress the urgency of climate action by invoking critical thresholds and feedback loops in the climate system (Russill and Nyssa 2009; van der Hel et al. 2018). One concept has been at the center of these efforts — the concept of “climatic tipping points” (Russill and Nyssa 2009; Russill 2015). Recent findings (e.g., Wunderling et al. 2021) have reignited the public debate surrounding tipping points and the risk of abrupt climate shifts. Surprisingly, however, there is a severe lack of systematic scientific studies on the effects of tipping point forewarnings on laypeople’s perceptions of climate change. As of now, there are no evidence-based guidelines available on how communicators should discuss tipping points in public settings. The present study aims to address this research gap and serves as a first step towards a better understanding of the socio-psychological effects of communication strategies that highlight the risk of (non-)linear shifts in the climate system.

### The tipping point metaphor

Tipping points are “critical threshold[s] at which a tiny perturbation can qualitatively alter the state or development of a system” (Lenton et al. 2008, p. 1786). The Greenland ice sheet and the Amazon rain forest are examples of climatic tipping elements that could qualitatively change their current state within the next century (Boers and Rypdal 2021; Lenton et al. 2008; Staal et al. 2020). Passing certain tipping points could lead to relatively abrupt changes in the climate system, which would have drastic and potentially irreversible impacts (Alley et al. 2003; Lenton et al. 2008; Mitchell et al. 2006; Steffen et al. 2018). While it was initially estimated that tipping points could be passed when average global temperatures rise above 5 °C relative to preindustrial levels, recent climate reports conclude that critical thresholds might be reached much earlier, at a warming of 1–2 °C (IPCC 2018, 2019; see also Armstrong Mackay et al. 2022). Several tipping points in the climate system might therefore be “dangerously close” (Lenton et al. 2019, p. 592) or might have already been triggered (Jansen et al. 2020).

Although considerable uncertainty remains regarding tipping elements and their sensitivity to global warming (e.g., Wunderling et al. 2021), the potential of abrupt changes in the climate system has been brought forward as a decisive reason to take swift and comprehensive action on climate change (e.g., Alley et al. 2003; Cai et al. 2016; Lenton and Schellnhuber 2007). For instance, Lenton et al. (2019) argued that “[…] the consideration of tipping points helps to define that we are in a climate emergency and strengthens […] calls for urgent climate action” (p. 592).

Science communicators have generally agreed that the concept of tipping points is a powerful metaphor in social discourses (Gardiner 2009; Nuttall 2012; Russill 2008, 2015; Russill and Nyssa 2009). Tipping point forewarnings tend to subvert expectations about climate change dynamics by implying that the Earth’s climate system is “much more sensitive to changes than commonly thought […]” (Russill and Nyssa 2009, p. 343). In fact, studies have indicated that laypeople often perceive climate change as a slow, gradual, and cumulative phenomenon, which will harm human and natural systems over a longer period of time (Fox-Glassman 2015; Milkoreit 2015; Sterman and Booth Sweeny 2002, 2007; Sterman 2011). This “static” mental model of climate change does not account for the potential of dynamic, nonlinear shifts in the climate system and might thus provoke misevaluations of the risks that arise from small increases in global temperatures (e.g., Sterman 2011; van Beek et al. 2022). Specifically, climate change might be perceived as more predictable and controllable than it actually is (Weber 2006).

Tipping point forewarnings, on the other hand, explicitly question the premise that global temperatures increase at a slow, steady, linear pace, provided greenhouse gas emissions are not reduced. By invoking the concept of tipping points, climate change is portrayed as a *non*linear and potentially abrupt phenomenon. In this sense, tipping point forewarnings encourage audiences to adapt their mental models of climate change to take the potential of nonlinear climate shifts into account (Russill and Nyssa 2009). As a result, laypeople might re-evaluate the risks associated with climate change; their risk judgments would consequently reflect a more differentiated and scientifically accurate understanding of climate dynamics.

In stark contrast, other scholars have viewed strategies that invoke tipping points and the risk of nonlinear climate shifts critically, arguing that these would be alarmist and ineffective (Bellamy and Hulme 2011; Hulme 2008; O’Neill et al. 2010, see also Gardiner 2009). For instance, O’Neill et al. (2010) associated tipping point forewarnings with a common framing of climate change as a “prospective environmental catastrophe” (p. 997). According to this line of reasoning, a nonlinear portrayal of climate change could induce anxiety and feelings of helplessness and might, in turn, negatively affect efficacy beliefs (Bellamy and Hulme 2011; O’Neill et al. 2010).

To our knowledge, there is currently no robust empirical evidence on whether communicating climate change as a nonlinear process is either effective or ineffective in heightening people’s climate change risk perceptions. Likewise, there are no conclusive findings on how nonlinear climate change portrayals affect efficacy beliefs and people’s willingness to counteract climate change, for instance, by donating money to an environmental organization. The present study addresses this research gap and constitutes a first attempt to systematically analyze how the communication of nonlinearities in the climate system affects people’s perceptions of climate change, especially judgments of risk.

### Public perceptions of nonlinear climate change

System dynamics studies have repeatedly demonstrated that laypeople tend to neglect or underestimate dynamic processes in the climate system (e.g., accumulation, feedback loops), which has led researchers to assume that mental models of climate change are mostly static (Sterman and Booth Sweeny 2002, 2007; Sterman 2011; see also van Beek et al. 2022).^1^ Static mental models of climate change have been associated with a lower sense of urgency and an illusion of control (e.g., Sterman 2011; Weber 2006). Lenton et al. (2008) noted that “[s]ociety may be lulled into a false sense of security by smooth projections of global change” (p. 1792), indicating a need for an increased awareness of climatic tipping points in order to promote proactive responses to climate change.

There are only few empirical studies that have explicitly examined perceptions of tipping points and nonlinear shifts in the climate system (e.g., Bellamy and Hulme 2011; Lowe et al. 2006; Milkoreit 2015). For instance, Bellamy and Hulme (2011) studied perceptions of risk related to abrupt climate changes and demonstrated that these perceptions were associated with worldviews derived from cultural theory (Douglas 1970). In a recent study, van Beek et al. (2022) evaluated the impact of a simulated climate negotiation task (“Tipping Point Negotiations”), designed to enhance the understanding and awareness of the threat of tipping points in participants of a climate conference. Van Beek et al. (2022) found that the intervention reduced the psychological distance of climatic tipping points. Qualitative analyses furthermore suggested that the general level of concern about climate change increased over the course of the intervention. The results indicate that raising awareness of climatic tipping points could indeed change perceptions of risk related to climate change.

Another stream of research has evaluated the impact of the disaster movie *The Day After Tomorrow* on public climate change perceptions and attitudes (Leiserowitz 2004; Lowe et al. 2006). The movie depicts abrupt, drastic shifts in the climate system as a result of anthropogenic climate change. In a field experiment, Lowe et al. (2006) found that climate change concern and action intentions increased after participants watched *The Day After Tomorrow*. Other studies that employed different methodological designs reached similar conclusions (Leiserowitz 2004; Reusswig 2005). Furthermore, there was a relatively consistent trend across studies that mental representations of the climate system shifted, in that participants perceived the climate system as less stable after watching the movie (Reusswig 2005).

Follow-up studies produced inconsistent results (e.g., Lowe 2006), questioning the assumption that the presentation of fictional and real-world “tipping point scenarios” might influence perceptions of climate change. One factor that could explain discrepancies in previous results is uncertainty related to predictions of nonlinear temperature increases. If tipping point scenarios are presented as fictional, inherently uncertain, or virtually impossible, the message might not necessarily be interpreted as relevant for climate change risk judgments (e.g., Lorenzoni et al. 2007; Spence et al. 2012). This would imply that a general awareness of the potential of abrupt, nonlinear climate shifts might not be sufficient to affect assessments of climate change risks *if* these events are considered extremely unlikely.

However, even if tipping point scenarios are presented as realistic, some audiences might still not respond to the message because of their pre-existing beliefs about climate change. For instance, if an audience is already highly alarmed by climate change, the presentation of tipping point scenarios might not elicit any cognitive or behavioral reaction. In fact, for some audiences, this might be the “default scenario” that they have been presented with most frequently in the past. Those who are highly alarmed by climate change might instead respond to a different scenario, which depicts future temperature increases as linear or gradual. A linear climate change portrayal could explicitly activate pieces of information that make the threat seem more controllable and less urgent (e.g., Weber 2006), which might, in turn, lead to a *decrease* in overall risk perceptions.

To understand how specific portrayals of climate change affect risk judgments, it seems indicated to compare the effects of nonlinear predictions of future temperature increases with the effects of linear predictions. Considering the limitations of previous research, it furthermore appears reasonable to control the level of prediction uncertainty to avoid interfering influences.

## The present research

The present study explicitly investigated the effects of different portrayals of climate change on intuitive judgments of risk and mental models, as well as people’s ensuing climate change mitigation behavior. In a preregistered, high-powered experiment, we tested the hypothesis that messages which portray climate change as a nonlinear, abrupt phenomenon provoke higher climate change risk judgments compared to messages presenting climate change as a gradual, linear phenomenon. Drawing on concepts from both cognitive and affective models of risk perception (Loewenstein et al. 2001; Slovic et al. 1980; Vlek and Stallen 1981), we developed a theoretical framework that proposes two pathways through which different climate change portrayals impact perceptions of risk. We predicted that nonlinear portrayals of climate change would affect judgments of risk by (a) re-structuring mental representations of climate change (i.e., cognitive pathway) and (b) intensifying negative affect associated with climate change (i.e., affective pathway).

If climate change is portrayed as a nonlinear, potentially abrupt process, we expected that certain qualitative risk characteristics become salient. Specifically, we predicted that climate change would be increasingly viewed as a risk with catastrophic and potentially uncontrollable consequences. Based on empirical and theoretical work linking the *perceived catastrophic potential* and the *perceived controllability of adverse consequences* of a hazard to judgments of risk (e.g., Marris et al. 1997; Nordgren et al. 2007; Slovic et al. 1980), we hypothesized that these changes in people’s mental representation of climate change will result in an increase in holistic risk perceptions (cognitive pathway).

Drawing on research on the interplay between affect and risk perception (Loewenstein et al. 2001; Slovic et al. 2004), we furthermore propose that nonlinear portrayals of climate change would provoke affective reactions strong enough to initiate a re-evaluation of climate risks. Previous studies have suggested that the confrontation with the risks of abrupt, nonlinear climate changes can induce fear and other negative affective associations (e.g., vivid mental imagery; see Bellamy and Hulme 2011; Lowe et al. 2006). Given that negative affect is a major precursor of climate change risk perceptions (van der Linden 2015), we predicted that negative affect would, in turn, lead to an increase in climate change risk perceptions (affective pathway).

### Hypotheses

**Fig. 1:**
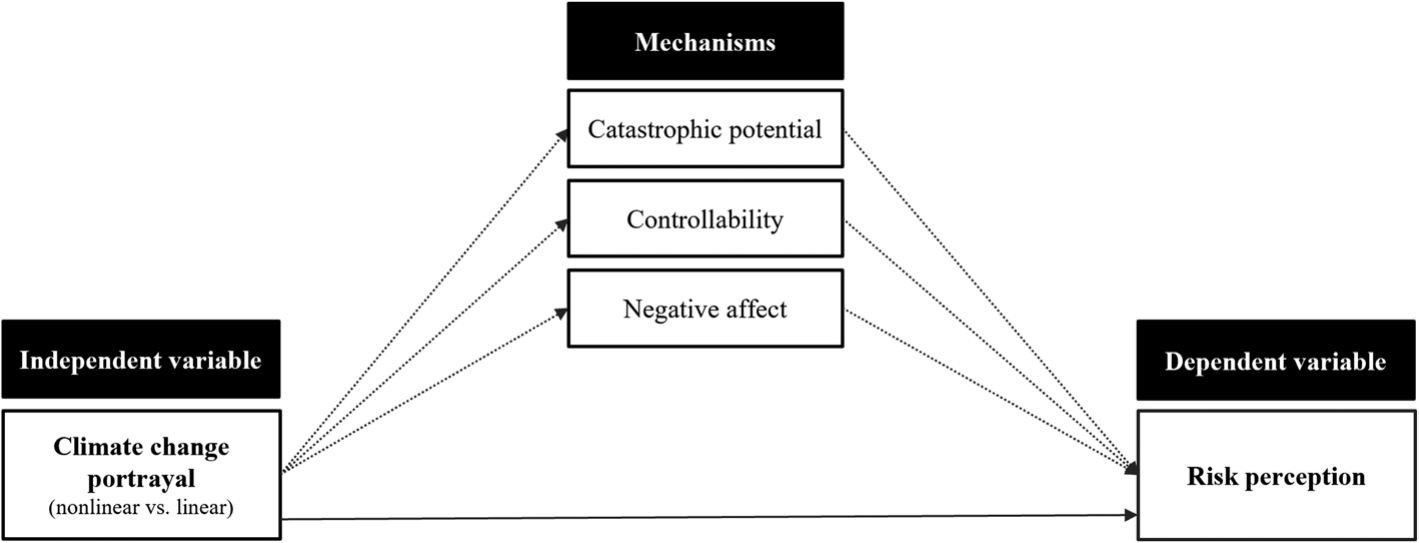
Proposed conceptual framework

Figure 1 shows the conceptual framework for the present study. Based on theoretical models of risk and research on the relationship between risk characteristics, affect and risk perceptions (Nordgren et al. 2007; Slovic et al. 1980; van der Linden 2015; Vlek and Stallen 1981), we predicted that a nonlinear (vs. linear) portrayal of climate change would provoke higher climate change risk perceptions (hypothesis 1). Furthermore, we expected that this effect would be mediated by the (1) perceived catastrophic potential, (2) perceived controllability of consequences, and (3) negative affect (hypothesis 2).

## Method

### Design

**Fig. 2:**
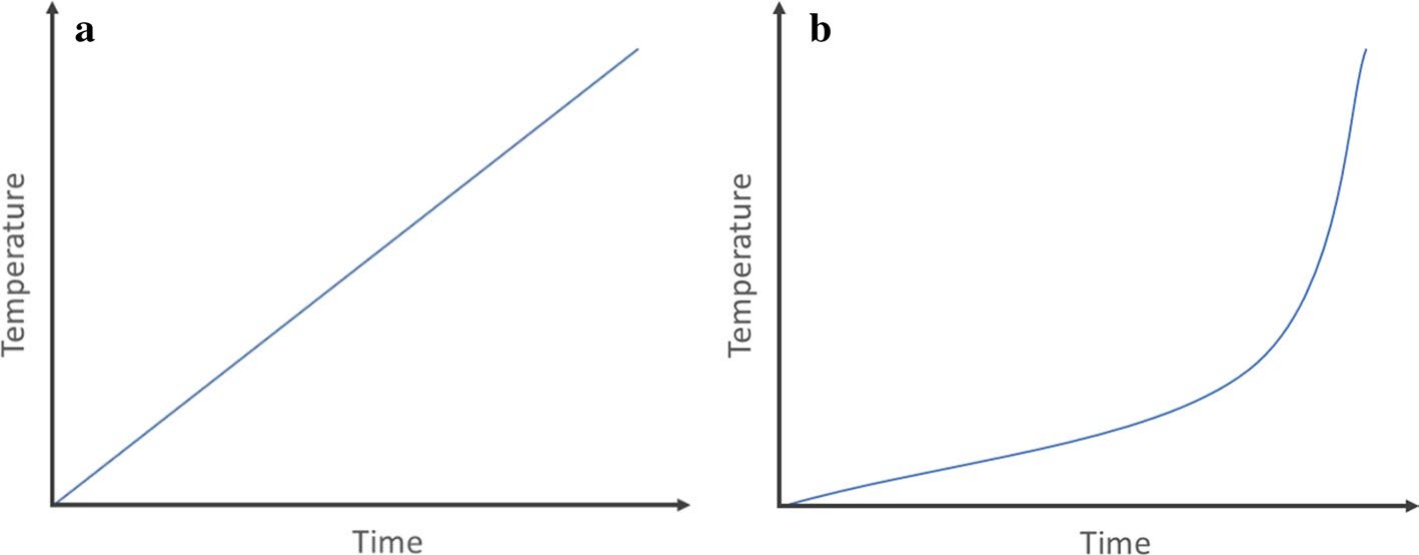
Linear (**a**) and nonlinear (**b**) development of global temperatures

The experimental design included one between-subjects factor with four conditions varying the *type of climate change portrayal* (linear vs. nonlinear vs. unspecified threat vs. no-message baseline). Participants were randomly assigned to the experimental conditions. In the linear and nonlinear portrayal condition, participants were exposed to a short message that either presented unmitigated climate change as a static, linear, or dynamic, nonlinear phenomenon. Additionally, these participants were provided with a graph that was said to depict future increases in global temperatures that would be observed if no mitigating actions were taken. The graph showed either a gradual, linear, or nonlinear, exponential increase in global temperatures (see Fig. 2). Participants in the unspecified portrayal condition, an active control condition, read the introductory part of the message that participants in the linear and nonlinear portrayal conditions received. This part merely outlined key facts about climate change without specifying the trajectory of future temperature increases. Accordingly, participants in the unspecified portrayal condition were not provided with a graph either. Participants in the no-message baseline condition (passive control group) did not receive any message or materials. The main dependent variable, climate change risk perception, and the proposed mediating variables were measured right after the participants were exposed to the stimulus materials or, in the baseline condition, after the participants completed the initial demographic questionnaire. We subsequently assessed a number of exploratory variables — in particular, efficacy beliefs and people’s willingness to donate money to an environmental organization that seeks to mitigate climate change.

The experiment was pre-registered (aspredicted.org). The preregistration, data set, and all experimental materials are available at https://osf.io/w6e5f/.

### Participants

An a priori power analysis was conducted to determine the sample size for this experiment. An exploratory study by van Beek (2018) found that an intervention to promote the understanding of tipping points and nonlinearities in the climate system produced a medium-sized effect (*d* = 0.5) on perceptions of risk related to abrupt climate change. Due to presumed differences in the intensity of the manipulation used in the current study and the study by van Beek (2018), a small-to-medium effect size (*d* = 0.35) was chosen as an input parameter for the power analysis (see also “safeguard power” analysis; Perugini et al. 2014). The analysis suggested that a total sample size of at least *N* = 360 was required to achieve a power of 0.80 (*α* = 0.05; G*Power 3.1, Faul et al. 2007). A total of 398 participants were recruited in social media groups, via e-mail distribution lists, and through the recruitment service *SurveyCircle*. Participants were told that they would be taking part in an online study on “science communication.” As compensation, participants received survey points (if required) and were furthermore invited to take part in a lottery with the prospect of winning a € 50 voucher of their choice. The data collection started in June 21 and ended on July 13, 2020.

### Procedure

After participants gave their consent to participate, they completed a short questionnaire with demographic items. Participants in the linear, nonlinear, and unspecified portrayal condition were subsequently presented with a short text, which they were asked to read thoroughly. It was announced that they would be expected to answer questions regarding the content of the text afterwards. In all conditions, the text was presented for at least 30 s. A “continue” button then appeared below the text, and participants were able to move on to the next part of the study.

In the linear, nonlinear, and unspecified portrayal condition, participants first read a short summary of key facts on anthropogenic climate change (see supplementary online materials). The experimental manipulation was implemented in the second paragraph of the text. This paragraph characterized future changes in the climate system as linear, nonlinear, or unspecified.

In the *linear* portrayal condition, participants were presented with the following paragraph:

[…] If the level of greenhouse gas emissions remains unchanged, there will be continuous changes in the global climate. That is, there will be a constant increase in global temperatures; as a result of this development, the Earth’s climate will change considerably. There is the risk that these changes will put enormous demands on or might even exceed the adaptive capacity of human civilization and natural systems.

Participants in the *nonlinear* portrayal condition read a similar paragraph that characterized potential future changes in the climate system as abrupt and dynamic instead:

[…] If the level of greenhouse gas emissions remains unchanged, there will be abrupt changes in the global climate as soon as global warming passes certain thresholds. That is, tipping points in the climate system will be reached; as a result of this development, the Earth’s climate will change considerably. There is the risk that these changes will put enormous demands on or might even exceed the adaptive capacity of human civilization and natural systems.

In the unspecified portrayal condition, participants were presented with the following statement instead:

There is the risk that the impacts of climate change will put enormous demands on or might even exceed the adaptive capacity of human civilization and natural systems.

Both messages portraying climate change as a linear or nonlinear phenomenon specifically referred to changes that would occur if global greenhouse gas emissions remained at a consistently high level (i.e., “business-as-usual scenario”), which allows for the possibility that consequential linear or nonlinear changes in the climate system could still be avoided.

After they had read the message, participants in the linear and nonlinear portrayal condition were informed that they would now be provided with a graph. The graph, which was presented on the following page, depicted the *average global temperature* as a function of the *time*. Participants were told that the graph illustrated how the Earth’s climate would change if the level of global greenhouse gas emissions remained unchanged.

The graph was designed to be as abstract and simple as possible: In the linear portrayal condition, the graph showed a linear increase in global temperatures over time (Fig. 2a), whereas, in the nonlinear condition, the graph showed a moderate, but accelerating increase that resembled an exponential function (Fig. 2b).

The nonlinear function illustrated a relatively sudden increase in global temperatures that could be observed when tipping points are passed and global warming is amplified due to feedback processes (see Lenton et al. 2008). Each line representing the increase in global temperatures was animated to appear successively, starting from the left-hand side. Participants started the animation manually by clicking on a “play” button. First, a coordinate system without any graphical content appeared. After 2 s, a line started to emerge from the zero point of the coordinate system, moving to the right-hand side at a steady rate. Within 6 s, the entire function was visible. After the animation had stopped, the function was presented for another 7 s before a “continue” button appeared below the diagram and participants were able to move on to the next part of the study.

To check whether participants had indeed read the text message, they were presented with a number of statements and were asked to indicate whether these were part of the text they had read before. All statements had in fact been part of the message participants were exposed to. Before moving on to the post-questionnaire, participants in the experimental conditions were additionally asked to answer three questions regarding (superficial) characteristics of the text message (e.g., readability). These items were used to disguise the real purpose of the experiment and to avoid *demand effects* (e.g., McCambridge et al. 2012).

After working through the text, materials, and control questions, participants then received the post-questionnaire containing the scales used to measure climate change risk perceptions and the proposed mediating variables (perceived catastrophic potential, perceived controllability, negative affect; see Fig. 1). The order of the two main parts of the questionnaire (i.e., risk perception, mediating variables) was counterbalanced. In the remaining part of the questionnaire, several exploratory variables were measured (e.g., perceived efficacy, donation behavior). The questionnaire also contained a measure of the perceived likelihood of abrupt changes in the climate system, given that global greenhouse gas emissions were to remain at a high level. This measure was used as direct manipulation check to assess whether the experimental treatment had an effect on people’s perceptions of climate dynamics. The questionnaire ended with several control questions, measuring participants’ belief in anthropogenic climate change, political ideology, and prior familiarity with the concept of climatic tipping points. Participants in the three experimental conditions were also asked to rate the perceived credibility of the text they had read. After completing the questionnaire, subjects were thanked for their participation and were debriefed.

We used the online platform Gorilla Experiment Builder (www.gorilla.sc) to create and host the study (Anwyl-Irvine et al. 2020).

### Measures

To measure the following dependent variables, we used established inventories, whenever available. When necessary, items were translated into German and adapted to the socio-cultural and political context in Germany. A full list of verbatim items is available on the accompanying OSF project.

#### Risk perception

*Climate change risk perception* was measured using a scale developed by van der Linden (2015), which consists of eight items (e.g., “How concerned are you with climate change?”). All items were answered on a 7-point scale (e.g., 1 = not concerned at all, 7 = very concerned). The scale distinguishes between *perceived societal* and *personal risk*, two interrelated dimensions (van der Linden 2015). Accordingly, three indices were created, a *societal* risk perception index (*α* *=* 0.84), a *personal* risk perception index (*α* = 0.85), and a *holistic* risk perception index (*α* *=* 0.89).

#### Psychological mediators

Drawing on previous work by Fischhoff et al. (1978), two items were developed to measure the *perceived catastrophic potential of climate change* (“Is climate change a risk that takes many lives at once [catastrophic] or only one at a time [chronic]?”, “Is climate change a risk that affects many people at once [catastrophic] or only one at a time [chronic]?”; 1 = chronic to 7 = catastrophic). The composite index had a relatively low internal consistency (Spearman-Brown *ρ* = 0.56). On a conceptual level, the moderate relationship between the two items could indicate that there is a difference in the expected distribution of *fatal* and *non-fatal* consequences of climate change over time. To account for potential conceptual differences, the two items were treated separately in the data analysis. However, as the main conclusions did not vary depending on whether the items were combined or separately analyzed, only the results for the composite index of *perceived catastrophic potential* are reported.

Building on previous work by Nordgren et al. (2007) and Fischhoff et al. (1978), two items were developed to measure the *perceived controllability of climate change* (“How much control would we have over climate change if greenhouse gas emissions were not reduced?”, “How much control would we have over climate change if planned climate protection measures were not implemented?”; 1 = no control at all; 7 = full control). The two items were highly correlated (*r* = .71, *p* < .001). They were combined into an index capturing the *perceived controllability of climate change* (Spearman-Brown ρ = 0.83).

*Negative affect* was measured with a three-item scale that was adapted from van der Linden (2015). The items capture broad affective evaluations of climate change (e.g., “To me, climate change is something…”; 1 = very bad, 7 = very good). A combined index of negative affect was created (*α* = 0.91), with higher values indicating higher negative affect.

#### Efficacy beliefs

*Perceived efficacy* in the context of climate change was measured using separate scales developed by van Zomeren et al. (2010) that capture two dimensions of the construct: *self-efficacy* and *collective efficacy*. *Self-efficacy* was measured with five items (e.g., “My individual actions will contribute to a solution of the climate crisis”; 1 = strongly disagree, 7 = strongly agree; *α* = 0.91). Three items assessed participants’ *collective efficacy* (e.g., “To what extent do you think that people can jointly prevent the negative consequences of the climate crisis?”; 1 = not at all, 7 = entirely; *α* = 0.82).

#### Donation behavior

*Donation behavior* was measured using a procedure developed by Clements et al. (2015). Participants were asked whether they wanted to participate in a lottery, with the prospect of winning a € 50 online voucher of their choice. They were informed that they could donate a share of the value of that voucher (0–50 €) to an environmental organization that is seeking to combat climate change. In case they were to win the lottery, the selected amount of money would be subtracted from the voucher that participants received. Participants could choose to donate to one of four German environmental organizations: *Friends of the Earth Germany*, *Fridays For Future Germany*, *Greenpeace Germany*, or *atmosfair*. Participants selected the amount of money they wanted to donate on a slider ranging from 0 to 50 €.

#### Manipulation check

One item was developed to measure the *perceived likelihood of abrupt climate shifts* as a result of unmitigated climate change (“How likely are abrupt changes in the climate system if global greenhouse gas emissions were not reduced?”; 1 = very unlikely, 7 = very likely).

Additionally, two items were used to assess the *perceived credibility of the text message* (e.g., “I considered the text message to be credible”, Spearman-Brown *ρ* = 0.70; 1 = strongly disagree, 7 = strongly agree).

#### Attention checks

 The questionnaire included three *attention checks*. The first attention check was conducted right after the introductory remarks had been presented. Participants were instructed to ignore the text of two subsequent questionnaire items and enter a non-related word (“lesen”) in both response boxes. The second and third attention check were integrated into the questionnaire itself; participants were asked to choose response option “2” and option “6,” respectively (scales ranging from 1 to 7). We excluded the data from participants who failed the second and/or third attention check (*n* = 17), resulting in a final sample of *N* = 381 (see Table 1 for a demographic description of the sample). Due to irregularities in the visual presentation of the first attention check, we decided to include the data from participants irrespective of their answers on this task. Follow-up analyses showed that the main results of the data analysis did not vary depending on these inclusion criteria.

**Table 1:** Demographic variables

Variable		Frequency (%)
Gender	Male Female Diverse	31.1 % 68.6 % 0.3 %
Age	14–19 20–29 30–39 40–49 50 ≤	6.4 % 64.7 % 12.0 % 4.3 % 12.6 %
Nationality	German Other	95.8 % 4.2 %

## Results

### Manipulation check

79.34% of all control questions about the content of the text messages were answered correctly, indicating that participants who had received a text message in the first part of the study had processed and understood the message. The text messages were furthermore perceived as credible across all conditions as indicated by an overall mean value that was significantly different from the scale mean of 4 (i.e., *M* = 5.79, *SD* = 0.96, *t*_one−sample_(256) = 30.01, *p* < .001, *d* = 1.87). A one-way analysis of variance (ANOVA) indicated that there were no significant differences in the perceived credibility of the text messages between the experimental conditions (*F*(2, 254) = 0.842, *p* = .43).

Importantly, the data also suggested that the experimental manipulation was successful in changing perceptions of climate dynamics. A one-way ANOVA revealed significant differences between the experimental conditions in the perceived likelihood of abrupt climate shifts as a result of unmitigated climate change (*F*(3, 373) = 8.33, *p* < .001, *η*^2^ = 0.06). Bonferroni tests indicated that participants in the nonlinear condition perceived abrupt climate shifts as more likely (*M* = 5.57, *SD* = 1.29) than participants in the linear portrayal condition (*M* = 4.81, *SD* = 1.50, *p* = .007, *d* = 0.54), and participants in both the active control condition (*M* = 4.49, *SD* = 1.55, *p* < .001, *d* = 0.76), and the passive control condition (*M* = 4.79, *SD* = 1.63, *p* = .003, *d* = 0.53). There were no significant differences between the linear condition and both the active and the passive control condition (*p*s > 0.90).

### Confirmatory analyses

#### Climate change risk perception

The mean risk perception scores were relatively high across all conditions (see Table 2), indicating that participants generally perceived climate change as a serious threat. Overall, the between-group variation in risk perception scores was quite small. A one-way ANOVA found that there were no significant differences in holistic risk perception scores across the four conditions (*F*(3, 377) = 0.08, *p* = .97). Thus, H1 was not supported — the type of climate change portrayal did not affect risk perceptions. Bayesian analyses corroborated this null finding, yielding empirical evidence for the absence of an effect (rather than absence of evidence). The BF_01_ = 80.86 showed strong evidence for the null hypothesis (see common Bayes conventions, Jeffreys 1961).

**Table 2:** Means (standard deviations) of dependent variables by experimental condition

	Nonlinear portrayal (*n* = 86)	Linear portrayal (*n* = 87)	Unspecified portrayal (*n* = 99)	No-message baseline (*n* = 109)	Entire sample (*n* = 381)
Risk perception	5.67 (0.90)	5.64 (0.84)	5.62 (0.82)	5.62 (0.93)	5.63 (0.87)
Mediators					
Catastrophic potential	2.84 (1.48)	2.72 (1.49)	2.73 (1.14)	2.71 (1.15)	2.75 (1.31)
Controllability of consequences	1.94 (0.67)	1.93 (0.79)	2.10 (0.92)	1.99 (0.90)	2.00 (0.83)
Negative affect	6.36 (0.67)	6.28 (0.93)	6.34 (0.78)	6.24 (0.92)	6.30 (0.83)

All variables were measured on 7-point scales, higher values reflect more of the construct

#### Perceived catastrophic potential

In general, participants perceived climate change as a chronic rather than a catastrophic risk. Participants assumed that climate change would harm or kill people over a longer rather than a shorter period of time (*M* = 2.75, *SD* = 1.31), *t*_one−sample_(380) = − 18.72, *p* < .001, *d* = − 0.96). A one-way ANOVA found no significant differences in the perceived catastrophic potential of climate change across the four conditions (*F*(3, 377) = 0.18, *p* = .91). Again, Bayesian analyses yielded empirical evidence for the null hypothesis (BF_01_ = 71.10). Thus, participants exposed to a nonlinear portrayal of climate change did not perceive the catastrophic potential of climate change as higher than participants exposed to a linear portrayal.

#### Perceived controllability of consequences

Across all conditions, participants indicated that they perceived the unmitigated consequences of climate change as uncontrollable rather than controllable (*M* = 2.00, *SD* = 0.83) (*t*_one−sample_(380) = − 47.09, *p* < .001, *d* = − 2.41). A one-way ANOVA revealed no significant differences in the perceived controllability of consequences between the four experimental conditions (*F*(3, 377) = 0.86, *p* = .46). The corresponding Bayes factor again empirically supported the null hypothesis (BF_01_ = 28.87). Hence, the results suggest that the nonlinear portrayal of climate change did not lead to an impaired perception of control over climate change consequences, relative to a linear portrayal of climate change.

#### Negative affect

In our sample, climate change was associated with substantial negative affect (*M* = 6.30, *SD* = 0.83) (*t*_one−sample_(380) = 53.97, *p* < .001, *d* = 2.77). A one-way ANOVA found no significant differences in negative affect between the four experimental conditions (*F*(3, 377) = 0.45, *p* = .72); Bayesian analyses again supported the null model (BF_01_ = 49.13). Thus, exposing participants to a nonlinear portrayal of climate change did not change their affective evaluation of the threat.

In sum, the experimental treatment did not significantly affect climate change risk perceptions, nor the proposed mediating variables — catastrophic potential, controllability of consequences, and negative affect. Therefore, hypotheses 1 and 2 were not supported.

### Exploratory analyses

#### Perceived efficacy

One-way ANOVAs indicated that there were no significant differences between the groups on both dimensions of perceived efficacy, self-efficacy (*F*_*self−efficacy*_(3, 366) = 0.971, *p* = .41) and collective efficacy (*F*_*collective efficacy*_(3, 366) = 1.055, *p* = .37).

#### Donations

**Fig. 3:**
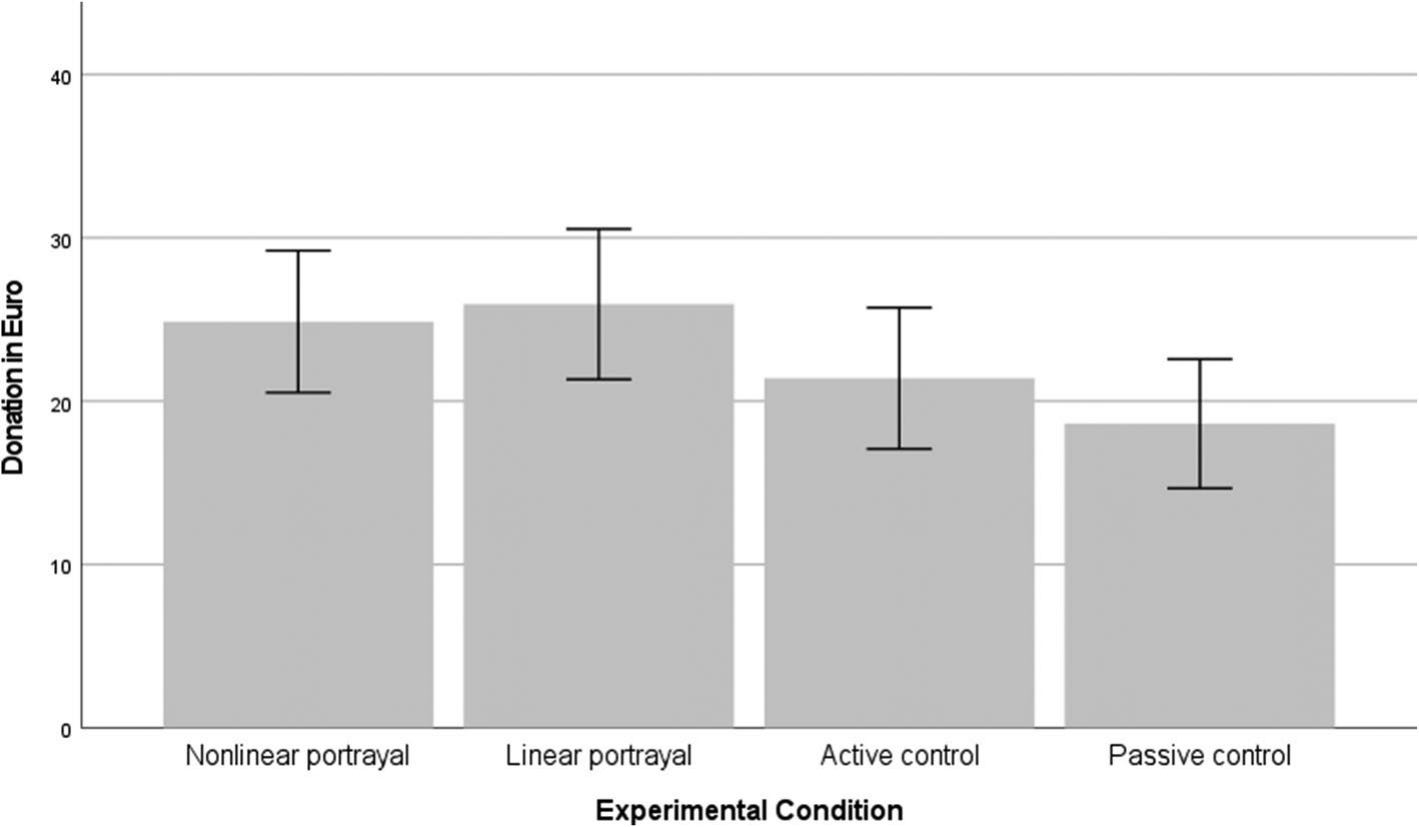
Average donation by experimental condition. Note: error bars represent 95% confidence intervals

In total, 252 of 381 participants (66.14%) donated at least 1 €, with 113 participants (29.66%) choosing to donate the maximum amount of 50 €. The average amount donated was *M* = 22.46 € (*SD* = 21.80). The frequency distribution of donation scores was u-shaped. A Kruskal-Wallis test of independent samples was performed to compare the central tendency of donation scores across the experimental conditions. The analysis produced a significant result (*H*(3) = 8.06, *p* = .045). Dunn’s pairwise tests revealed significant differences between the passive control condition and the linear portrayal condition (*p* = .013), and the nonlinear portrayal condition (*p* = .028). Thus, participants who received the full experimental treatment (nonlinear or linear portrayal of climate change) donated higher amounts of money — on average 7 € more — than participants in the passive control group (see Fig. 3). The linear and nonlinear condition did not differ (*p* = .782). In addition, no significant differences were observed between the active and the passive control condition (*p* = .354), or the active and the nonlinear or the linear portrayal condition (*ps* > 0.10) (see Fig. 3).

## Discussion

In the present study, we have investigated whether communication strategies that present climate change as a nonlinear phenomenon can increase laypeople’s perceptions of risk, compared to communication strategies that portray climate change as a linear process. In particular, we have put to a first experimental test the idea that highlighting tipping points and nonlinearities in the climate system might fundamentally change the way laypeople think about the threat of climate change (see Nuttall 2012; Russill and Nyssa 2009; van der Hel et al. 2018).

### Findings

Our study suggests that the type of climate change portrayal did not affect judgments of risk. While the perceived likelihood of abrupt climate shifts was increased by a nonlinear portrayal of climate change, no differences were observed between the experimental groups on negative affect or perceived risk characteristics such as the catastrophic potential of climate change and the controllability of consequences. Bayesian analyses yielded strong support for the respective null hypotheses. Thus, rather than offering “absence of evidence” due to a frequentist null finding, our Bayesian analyses offer “evidence of absence.” We discuss a number of potential reasons for these null findings below.

Our results call common assumptions regarding the effects of tipping point forewarnings into question. We found no support for the notion that tipping point forewarnings have a profound impact on judgments of risk related to climate change (cf. Lenton et al. 2008; Russill 2015). Based on our data, it is not evident that messages which invoke critical thresholds in the climate system elicit different social-cognitive reactions in laypeople than messages which describe climate change in linear terms. At the same time, however, our analysis also suggests that there was no *backfire effect* concerning efficacy beliefs (cf. Bellamy and Hulme 2011). Participants who were exposed to a nonlinear portrayal of climate change did not report lower levels of perceived efficacy than participants who were exposed to a linear portrayal. This might imply that nonlinear portrayals of climate change do not necessarily provoke feelings of helplessness and resignation in connection with climate change.

Further analyses of our data revealed another notable finding: The willingness to donate money to environmental organizations differed significantly across the experimental conditions. We identified a trend towards higher donations in the conditions that received the full experimental treatment, relative to the passive control group. Participants who were confronted with specific predictions concerning future temperature increases (linear *or* nonlinear) were willing to donate more money than participants who received no treatment at all. The results indicate that a specification of the trajectory of future temperature increases might help promote behavioral responses to the threat of climate change. These findings are in line with previous studies indicating that reducing uncertainty in climate change predictions might promote pro-environmental action intentions if the predicted outcomes are framed as losses (e.g., Morton et al. 2010).

### Limitations

Overall, the effect of our experimental treatment on perceptions, attitudes, and motivational dispositions related to climate change was small. To a certain extent, this could be attributable to the design of the experimental treatment. The experimental materials that we used were relatively abstract and ambiguous. Participants received rudimentary information concerning linear or nonlinear changes in the climate system that might be expected in the future. The materials did not include descriptions of specific tipping elements (e.g., Greenland ice sheet) or the feedback processes that could lead to nonlinear climate shifts. Furthermore, participants who were exposed to nonlinear predictions were not provided with estimates as to *when* tipping points might be passed (see Fig. 2). The only difference between the linear and nonlinear portrayal condition was the description of the nature of future temperature increases that would be expected if greenhouse gas emissions were not reduced.

However, this might not be enough to convey the personal and societal relevance of climatic tipping points. It is possible that our experimental manipulation did not enable participants to locate the threat of climatic tipping points and to establish connections between their previous knowledge on climate change and the new information that was presented. This would also explain why participants’ perceptions of the catastrophic potential and the controllability of climate change impacts appeared to be unaffected. Climate change is a geographically and temporally “un-situated risk” (Hulme 2009, p. 196), which is one reason why laypeople generally struggle to process and evaluate risk-related information on this threat (see also Helgeson et al. 2012). Furthermore, recent evidence suggests that laypeople might not intuitively understand the concept of tipping points in the context of climate change (Bruine De Bruin et al. 2021). Therefore, it might be even more crucial to provide temporal and geographical references when presenting “tipping point scenarios,” given the high complexity of this subject matter. For instance, risk communicators might stress that tipping points could be reached within the next decades, sooner than initially expected (e.g., Lenton et al. 2019). This could help audiences understand the significance of tipping points in concrete terms. At the same time, reducing temporal uncertainty might also counter the widespread tendency to be unrealistically optimistic about the effects of climate change (*optimism bias*, e.g., Gifford et al. 2009). If temporal references are provided, laypeople might be less likely to categorize nonlinear shifts in the climate system as a distant, harmless threat.

Although it might be strategically wise to make the threat of tipping points as concrete and tangible as possible, we shall not forget that there are still significant limitations to our scientific understanding of tipping elements and their sensitivity to global warming (e.g., Wunderling et al. 2021). In the present study, we have relied on a simplified conceptualization of climatic tipping points and we did not explicitly mention any uncertainty associated with nonlinear predictions of climate change. However, in public conversations about climatic tipping points, science communicators are well-advised to address the limitations of previous research in this field. Presenting predictions of nonlinear changes in the climate system as virtually certain would misrepresent the current state of research (e.g., Lenton et al. 2008).

Explicitly addressing uncertainties and limitations to our understanding of nonlinear climate shifts might affect how laypeople respond to the presentation of tipping point scenarios. Therefore, the extent to which our findings generalize to real-world contexts is unknown. Further research is necessary to understand the role of uncertainty in the context of tipping point forewarnings. The effects of uncertainty might also depend on the message framing: If science communicators stress that uncertainty might not be “working in our favor” and that high levels of uncertainty should encourage, not discourage, rapid decarbonization (e.g., Lewandowsky et al. 2014), then the perceived message relevance might not be undermined.

Another limitation of the present study concerns sample characteristics. Based on our results, it is difficult to rule out the possibility of attribute-treatment interactions. In our sample, in which female and college-educated individuals were strongly overrepresented, climate change was already considered a serious threat. Beyond these high baseline levels, the strategy of highlighting “tipping point scenarios” may have limited clout to produce additional shifts in overall risk perceptions for this audience that is already alarmed by climate change. To expand this reasoning, future studies could target specific subgroups of the population that are less concerned with climate change. Furthermore, it is indicated to evaluate the effects of nonlinear descriptions of climate change in strongly polarized socio-cultural environments where public attitudes might be governed by different political dynamics.

Finally, we need to discuss the potential influence of external, socio-historical factors on the present results. As the current study was conducted during a major public health crisis, the SARS-CoV-2 pandemic, there is reason to believe that members of the general public were more sensitive to public health risks than under previous circumstances. Furthermore, considering the role that the concept of exponential growth played in the context of recent public health campaigns (Podkul et al. 2020), it is reasonable to assume that public awareness of nonlinear, exponential functions and the behavior of complex, dynamic systems was unusually high, potentially at an unprecedented level. Therefore, it is possible that the information we provided about nonlinear developments was processed and interpreted differently than under usual circumstances and given the common pre-pandemic knowledge. In that sense, some of the reported null findings might represent true population null effects that have emerged over the past years. Put differently, the enhanced sensitivity for the concept of nonlinear change may have attenuated the response to our experimental treatment. However, based on the available data, it is difficult to determine the extent to which contextual factors influenced the processing of the risk-related information provided in the current study.

### Directions for future research

Interventions that rely on active learning strategies to raise awareness of climatic tipping points have produced promising results (e.g., van Beek et al. 2022). These studies indicate that interactive materials might help illustrate why proactive responses are necessary to avoid devastating and uncontrollable climate impacts. Future research could compare the effects of passive and active learning strategies on climate change perceptions and attitudes. As of now, and in light of the results of our present study, there is no conclusive evidence that interventions that rely on passive learning strategies to promote laypeople’s understanding of nonlinear climate shifts (e.g., text-based interventions) can successfully change attitudes and perceptions related to climate change (see also Lowe 2006). However, considering the possibility that communication-based interventions could reach more people in a shorter period of time as compared to other types of interventions (e.g., simulation games, workshops), it might be critical to develop strategies to effectively communicate the risks of nonlinear climate shifts.

Future research should also take a closer look at the psychological processes that are triggered by tipping point forewarnings. In the present study, we have proposed a relatively simple conceptual model with two distinct pathways through which nonlinear portrayals of climate change could impact risk perceptions — a cognitive and an affective pathway (Fig. 1). It should, however, be noted that cognitive and affective responses to nonlinear climate change portrayals may not be entirely independent from each other. For instance, it is conceivable that cognitive assessments of risk-related information initiate affective responses. This idea would be consistent with appraisal theory (e.g., Frijda et al. 1989; see also Yang et al. 2014). Given these considerations, we propose that a sequential mediation model should be used in future studies, as it takes potential causal sequences between mediating variables into account.

## Conclusion

The present study sought to systematically analyze how communication strategies that present climate change as a linear or nonlinear phenomenon affect perceptions of risk, attitudes, and motivational dispositions related to this threat. Overall, our results did not support frequent claims brought forward by science communicators about the effects of tipping point forewarnings. The present results, however, only allow for limited conclusions given the lack of a high-intensity treatment, the specific participant sample recruited, and the potential influence of the SARS-CoV-2 pandemic. Further research is necessary to address several unanswered questions. As the Earth’s climate system approaches critical thresholds and climate scientists gain more insights into climate dynamics, tipping points will likely receive more attention in future public discourses. Therefore, we argue that science communicators need evidence-based guidelines on how to discuss the threat of nonlinear climate shifts in public settings. We encourage behavioral scientists to address the research gap that we have identified in the field of climate change communication, in order to improve efforts to reach out to policymakers and the general public.

## Supplementary information

Below is the link to the electronic supplementary material.

ESM 1 (DOCX 60.1 KB)

## Data Availability

The data set generated during the current study is available at https://osf.io/w6e5f/.

## Footnotes

1 This assumption is also in line with the well-established finding that laypeople have general difficulties in understanding nonlinear relationships and the behavior of feedback systems (e.g., Wagenaar and Sagaria 1975).

## Materials availability

The materials that were used for the current study are available at https://osf.io/w6e5f/.

## Code availability

The SPSS Code is available at https://osf.io/w6e5f/.
