# Supplementary material for: Tipping points ahead? How laypeople respond to linear versus nonlinear climate change predictions
Source: Clim Change. 2022 Nov 21;175(1-2):8. doi: 10.1007/s10584-022-03459-z (PMC9676726; doi:10.1007/s10584-022-03459-z)
Supplement: Supplementary file 1 — (DOCX 60.1 KB) [file 10584_2022_3459_MOESM1_ESM.docx]

**Tipping points ahead? How laypeople respond to linear versus nonlinear climate change predictions**

Felix J. Formanski^1^, Marcel M. Pein^1^, David D. Loschelder^2^, John-Oliver Engler^3^, Onno Husen^4^ and Johann M. Majer^5^

^1^ Faculty of Sustainability, Leuphana University Lüneburg, Lüneburg, Germany

^2^ Faculty of Business and Economics, Leuphana University Lüneburg, Lüneburg, Germany

^3^ Faculty of Natural and Social Sciences, University of Vechta, Vechta, Germany

^4^ Faculty of Education and Social Sciences, University of Münster, Münster, Germany

^5^ Faculty of Education and Social Sciences, University of Hildesheim, Hildesheim, Germany

Correspondence concerning this article should be addressed to Felix J. Formanski or Johann M. Majer, E-mail: [felix.j.formanski@stud.leuphana.de](mailto:felix.j.formanski@stud.leuphana.de) or [majer@uni-hildesheim.de](mailto:majer@uni-hildesheim.de).

Journal: Climatic Change

Submission date: May 10, 2022

**Supplementary Online Materials**

1. **Experimental Treatment**

In the linear, nonlinear and unspecified portrayal condition, participants were informed that they would be presented with a short text:

*“On the next slide, you will see a short text. Please read this text carefully. Afterwards you will be asked to answer a number of questions about the text.*

*Take enough time to read the text. After you have finished, continue the study by clicking on ‘Next’.”*

In all conditions, the text began with a short summary of key facts on anthropogenic climate change:

*“Over the last 100 years, global temperatures have risen about 1°C due to increasing*

*emissions of greenhouse gases like carbon dioxide (CO2) and methane (CH4). This ongoing*

*warming will have widespread consequences for life on Earth. As a general principle, the*

*impacts of climate change will be more severe in the case of a high as compared to low*

*increase in global temperatures.”*

Participants in the unspecified portrayal condition additionally read the following

sentence:

*“There is the risk that the impacts of climate change will put enormous demands on or might*

*even exceed the adaptive capacity of human civilization.”*

In the linear portrayal condition, participants were confronted with the following

paragraph instead:

*“If the level of greenhouse gas emissions remains unchanged, there will be continuous*

*changes in the global climate. That is, there will be a constant increase in global*

*temperatures; as a result of this development, the Earth’s climate will change considerably.*

*There is the risk that these changes will put enormous demands on or might even exceed the*

*adaptive capacity of human civilization.”*

Participants in the nonlinear portrayal condition were presented with a similar

paragraph, that characterized potential future changes in the climate system as abrupt and

dynamic instead:

*“If the level of greenhouse gas emissions remains unchanged, there will be abrupt changes in*

*the global climate as soon as global warming passes certain thresholds. That is, tipping*

*points in the climate system will be reached; as a result of this development, the Earth’s*

*climate will change considerably. There is the risk that these changes will put enormous*

*demands on or might even exceed the adaptive capacity of human civilization.”*

After they had read the text, participants in the linear and nonlinear portrayal condition were informed that they would now be provided with additional material:

*“On the following slide, you will see a short video. The video illustrates how the Earth’s climate will change if global greenhouse gas emissions are not reduced.*

*This development will be visualized in a graph. The graph presents the time on the (horizontal) y-axis and the average global temperature on the (vertical) x-axis.*

*On the upcoming slide, please klick on the “play” button located in the middle of the screen. The video will then start after a few seconds.”*

1. **Measures**

2.1 Climate change risk perception scale (van der Linden 2015)

Table: Means (standard deviations) of risk perception items

|  | Mean (SD) |
| --- | --- |
| 1. How concerned are you about climate change? (1 = Not concerned at all, 7 = Very concerned) | 5.78 (1.12) |
| 2. How often do you worry about the potentially negative consequences of climate change (1 = Very rarely; 7 = Very frequently) | 5.15 (1.27) |
| 3. In your judgment, how likely are you, sometime during your life, to experience serious threats to your health or overall well-being, as a result of climate change? (1 = Very unlikely, 7 = Very likely) | 5.03 (1.50) |
| 4. How serious of a threat do you believe that climate change is, to you personally? (1 = Not serious at all, 7 = Very serious) | 4.88 (1.38) |
| 5. In your judgment, how likely do you think it is that climate change will have very harmful, long-term impacts on our society? (1 = Very unlikely, 7 = Very likely) | 6.32 (0.92) |
| 6. How serious of a threat do you think that climate change is to the natural environment? (1 = Not serious at all, 7 = Very serious) | 6.46 (0.87) |
| 7. How serious would you rate current impacts of climate change around the world? (1 = Not serious at all, 7 = Very serious) | 6.08 (0.95) |
| 8. How serious would you estimate the impacts of climate change for Germany? (1 = Not serious at all, 7 = Very serious) | 5.37 (1.08) |

Note: Items 1-4 measure the perceived personal risk; items 5-8 measure the perceived societal risk.

2.2 Measurement of perceived risk characteristics

Table: Means (standard deviations) of risk characteristics items

|  | Mean (SD) |
| --- | --- |
| Perceived catastrophic potential |  |
| 1. Is climate change a risk that takes many lives at once (catastrophic) or only one at a time (chronic)?” (1 = Chronic, 7 = Catastrophic) | 2.64 (1.43) |
| 2. Is climate change a risk that affects many people at once (catastrophic) or only one at a time (chronic)?” (1 = Chronic, 7 = Catastrophic) | 2.85 (1.70) |
| Perceived controllability of consequences |  |
| 1. How much control would we have over climate change if greenhouse gas emissions were not reduced?” (1 = No control at all, 7 = Full control) | 2.07 (0.92) |
| 2. How much control would we have over climate change if planned climate protection measures were not implemented? (1 = No control at all, 7 = Full control) | - 1. (0.88) |

2.3 Negative affect scale (van der Linden 2015)

Table: Means (standard deviations) of affect items

|  | Mean (SD) |
| --- | --- |
| 1. To me, climate change is something… (1 = Very positive, 7 = Very negative) | 6.34 (0.91) |
| 2. Overall, I feel that climate change is… (1 = Very favorable, 7 = Very unfavorable) | 6.31 (0.88) |
| 3. I see climate change as something… (1 = Very pleasant, 7 = Very unpleasant) | 6.26 (0.92) |

1. **Correlational analyses**

Table: Correlations between the dependent measures

|  | 1 | 2 | 3 | 4 | 5 | 6 | 7 | 8 |
| --- | --- | --- | --- | --- | --- | --- | --- | --- |
| Risk perception | 1.00 | 0.03 | -0.39^***^ | 0.51^***^ | 0.36^***^ | 0.45^***^ | 0.37^***^ | 0.26^***^ |
| Catastrophic potential | 0.03 | 1.00 | -0.06 | -0.14^**^ | 0.13^*^ | 0.04 | 0.001 | 0.01 |
| Controllability of consequences | -0.39^***^ | -0.06 | 1.00 | -0.31^***^ | -0.22^***^ | -0.17^**^ | -0.14^**^ | -0.12^*^ |
| Negative affect | 0.51^***^ | -0.14^**^ | -0.31^***^ | 1.00 | 0.19^***^ | 0.23^***^ | 0.18^**^ | 0.17^**^ |
| Likelihood of abrupt changes | 0.36^***^ | 0.13^*^ | -0.22^***^ | 0.19^***^ | 1.00 | 0.17^***^ | 0.18^***^ | 0.12^*^ |
| Self-efficacy | 0.45^***^ | 0.04 | -0.17^**^ | 0.23^***^ | 0.17^***^ | 1.00 | 0.47^***^ | 0.32^***^ |
| Collective efficacy | 0.37^***^ | 0.001 | -0.14^**^ | 0.18^**^ | 0.18^***^ | 0.47^***^ | 1.00 | 0.11^*^ |
| Donations | 0.26^***^ | 0.01 | -0.12^*^ | 0.17^**^ | 0.12^*^ | 0.32^***^ | 0.11^*^ | 1.00 |

**p* < 0.05, ***p* < 0.01, ****p* < 0.001

*Correlational analyses.* Among the variables considered in our conceptual framework, negative affect was the one most strongly associated with holistic risk perceptions. Negative affect was predictive of higher climate change risk perceptions (*r* = .51, *p* < .001). Furthermore, there was a significant negative relationship between the perceived controllability of consequences and risk perceptions (*r* = -.39, *p* < .001). Perceiving unmitigated climate change as an uncontrollable threat was thus associated with higher climate change risk perceptions. Contrary to our expectations, there was no significant relationship between the perceived catastrophic potential and risk perceptions (*r* = .03, *p* = .54). The perception of climate change as a chronic or catastrophic risk was, therefore, unrelated to overall judgments of the seriousness of the threat. However, further analyses revealed that there was a significant correlation between risk perceptions and the perceived likelihood of abrupt climate shifts. That is, participants who considered abrupt shifts in the climate system as more likely reported higher risk perceptions (*r* = .36, *p* < .001).

1. **Separate analysis using the data from participants who passed all three attention checks (*n* = 190)**

Table: Means (standard deviations) of dependent variables by experimental condition

|  | Nonlinear portrayal  (n = 41) | Linear portrayal  (n = 42) | Unspecified portrayal  (n = 47) | No-message baseline  (n = 60) | Entire sample (N = 190) |
| --- | --- | --- | --- | --- | --- |
| Risk perception: Holistic | 5.76 (0.86) | 5.79 (0.78) | 5.58 (0.85) | 5.49 (1.03) | 5.64 (0.90) |
| Catastrophic potential | 2.96 (1.41) | 2.63 (1.45) | 2.53 (1.06) | 2.62 (1.03) | 2.67 (1.23) |
| Controllability of consequences | 1.92 (0.63) | 1.93 (0.80) | 2.01 (0.59) | 2.01 (0.98) | 1.97 (0.78) |
| Negative affect | 6.43 (0.62) | 6.51 (0.66) | 6.31 (0.81) | 6.26 (0.86) | 6.37 (0.76) |

Note: Risk perception was measured on a 7-point scale, higher values indicate higher perceived risk; Catastrophic potential was measured on a 7-point scale (1 = Chronic, 7 = Catastrophic); Controllability of consequences was measured on a 7-point scale (1 = No control at all, 7 = Full control); Negative affect was measured on a 7-point scale, higher values indicate higher negative affect

Across the experimental conditions, there were no significant differences in holistic risk perception scores, *F*(3, 186) = 1.20, *p* = .31, in the perceived catastrophic potential, *F*(3, 186) = 1.03, *p* = .38, in the perceived controllability of climate change, *F*(3, 186) = 0.20, *p* = .90., or in negative affect, *F*(3, 186) = 1.06, *p* = .37.
